# The protective effect of serum carotenoids on cardiovascular disease: a cross-sectional study from the general US adult population

**DOI:** 10.3389/fnut.2023.1154239

**Published:** 2023-07-12

**Authors:** Min Wang, Renzhe Tang, Rui Zhou, Yongxiang Qian, Dongmei Di

**Affiliations:** Department of Cardiothoracic Surgery, The Third Affiliated Hospital of Soochow University, Changzhou, Jiangsu Province, China

**Keywords:** serum carotenoids, cardiovascular disease, coronary heart disease, lycopene, NHANES, WQS

## Abstract

**Background:**

Cardiovascular disease (CVD) has become a key global health issue. Serum carotenoids are associated with CVD, while their effects on different diseases remain unclear. Herein, the relationship between the concentration of serum carotenoid and the CVD risk was investigated using nationwide adult samples obtained from the USA.

**Materials and methods:**

Data of National Health and Nutrition Examination Survey (NHANES) in 2001–2006 were employed. The association of serum carotenoids (total, lycopene, β-carotene, α-carotene, lutein/zeaxanthin, and β-cryptoxanthin) with CVD was explored by using multivariate logistic, linear and weighted quantile sum (WQS) regression analyses. Eventually, data from 12,424 volunteers were analyzed for this study.

**Results:**

Multivariate model data showed that lutein/zeaxanthin, α-carotene, lycopene, and β-cryptoxanthin were negatively associated with the prevalence of CVD (*p* < 0.05). In comparison with the first quartile, the fourth quartile was associated with α-carotene ([OR] = 0.61 [0.47–0.79]), β-cryptoxanthin (OR = 0.67 [0.50–0.89]), lutein (OR = 0.69 [0.54–0.86]), and lycopene (OR = 0.53 [0.41–0.67]). WQS analysis revealed that the combination of serum carotenoids had negative correlation with the prevalence of total CVD (OR = 0.88, 95% CI: 0.85–0.92, *p* < 0.001). Additionally, dose–response analysis demonstrated a negative linear association of hypertension with all the carotenoids involved (*p* > 0.05 for non-linearity).

**Conclusion:**

The concentration of serum carotenoids had negative correlation with the prevalence of CVD, with a more significant negative effect against heart attack and stroke.

## 1. Introduction

CVD involves the blood vessels or heart and include congestive heart failure (CHF), coronary heart disease (CHD), rheumatic heart disease, heart attack, peripheral artery disease, angina, and stroke. Indeed, the CVD-caused death cases worldwide reached 17.8 million in 2019 and may increase to 23 million by 2030 (1, 2). Epidemiological evidence has suggested that the CVD risk is negatively correlated with the diet quality (3).

In terms of nutrition and health, some carotenoids can be used as vitamin A precursors, where α-carotene, β-carotene and β-cryptoxanthin can be converted to vitamin A. In daily diet, carotenoids can be obtained from lettuce, carrots, tomatoes and oranges (4). Carotenoids can be divided into hydrocarbon carotenoids and oxygen-containing derivatives of hydrocarbon carotenoids according to the chemical structure. Hydrocarbon carotenoids include lycopene, β-carotene, and α-carotene, and oxygenated derivatives of hydrocarbon carotenoids (e.g., xanthophylls, neoxanthin, violet xanthin, lutein, and β-cryptoxanthin) (5, 6). Carotenoids have antioxidant activity, which can prevent and treat CVD. In addition, carotenoids may be involved in cellular signaling pathways correlated with inflammation and oxidative stress (OS), thereby inhibiting OS and inflammation (7). It has been demonstrated that the total concentration of carotenoid in blood lower than 1,000 nmol/L is related to a high risk of chronic diseases (8). Epidemiological studies have shown that 2–20 mg of lycopene intake per day can effectively prevent CVD (9). The effects on atherosclerosis and hypertension are even more pronounced (9, 10). Nevertheless, some studies have delivered different conclusions. Specifically, the correlation of increased carotenoid intake and reduced CVD risk remains controversial. A non-linear dose–response meta-analysis showed that the risk of cardiovascular death did not change with increasing dietary β-carotene intake (11). However, β-carotene was associated with increased all-cause mortality in another study of beta carotene supplements, and one-time beta carotene use was positively associated with cardiovascular events. Hence, treatment by β-carotene alone is not recommended for CVD (12). Indeed, the use of carotenoids or vitamin E supplements to counteract CVD or carcinoma has been opposed (13, 14).

This study aims to investigate the correlation of the serum level of carotenoids with the CVD risk by using nationwide adult samples obtained from the USA. Specifically, data of National Health and Nutrition Examination Survey (NHANES) in 2001–2006 were utilized to assess the effect of serum carotenoids on CVD.

## 2. Materials and methods

### 2.1. Experimental design

The NHANES is a nationwide survey aiming to the evaluate nutritional status and health of the population. It was executed by the Centers for Disease Control and Prevention (CDC) of the USA. This study combined interview and physical examination (15). The interviews covered various fields, including health, diet demographic, and socioeconomic information. Informed consent was obtained from each participant and approval of the NHANES protocol was obtained from the NCHS Research Ethics Review Committee. From 2001 to 2006, a total of 31,509 individuals participated in NHANES. 9,331 of the 31,509 participants were excluded as they had no data on serum carotenoids. Meanwhile, participants aged below 18 years old were excluded. Additionally, pregnant participants were excluded. Eventually, data from 12,424 adults from NHEANES were included in this analysis (Supplementary Figure S1).

### 2.2. Measurement of carotenoid concentrations

Serum levels of β-carotene, lycopene, lutein/zeaxanthin, α-carotene, and β-cryptoxanthin were measured by using high performance liquid chromatography (HPLC) for NHANES 2001–2002 and 2005–2006, while the serum levels of six carotenoids were determined by utilizing a comparable HPLC method for NHANES 2002–2003. This method was performed based on multiwavelength photodiode absorbance (absorbance = 450 nm). NHANES 2003–2004 data were converted to equivalent carotenoid measurements by HPLC using a regression method. Total carotenoid concentrations in serum were determined based on the serum levels of the five carotenoids mentioned above. Protocols and quality control were designed accordingly (16, 17).

### 2.3. Determination of covariates

In the NHANES study, household interviews (using standardized questionnaire) and medical assessments were employed to collect data. Age, gender, education level, household income race, smoking and alcohol drinking history, physical activity, BMI, energy intake level, hyperlipidemia, diabetes and hypertension of the participants were obtained based on previous studies with confounding covariates for CVD.

In terms of race and ethnicity, the participants were grouped as “Mexican American,” “Other Hispanic,” “Non-Hispanic White,” “Black “and “other.” In terms of education levels, the participants were grouped as “less than high school,” “high school,” and “high school and above.” Poverty was determined based on the household income-to-poverty ratio (household income-to-poverty ratio < 1 indicates poverty). In terms of smoking history, participants who smoked less than 100 cigarettes during lifetime were categorized as never smokers, participants who smoked over 100 cigarettes during lifetime were categorized as current smokers, and participants who smoked more than 100 cigarettes but had quit were categorized as former smokers. In terms of drinking history, the participants were grouped as ‘no drinking’, ‘low to moderate drinking’ (less than two drinks and one drink daily for the male and the female, respectively), and ‘heavy drinking’ (more than two drinks and one drink daily for the male and the female, respectively). The energy intake was defined as the average of dietary intake in 2 days. In terms of physical activity, the participants were grouped as ‘inactive’, ‘insufficiently active’ and ‘active’. Hypertension and diabetes were identified based on self-reported histories of physician-diagnosed hypertension (yes or no), physician-diagnosed diabetes (yes or no), anti-hypertensive medications (yes or no), and anti-hyperglycemic medications (yes or no).

### 2.4. Statistical analysis

National estimates were effectively generated using weighted analyses, and CDC guidance was followed with adjustment for over-sampling of minority subgroups. Continuous and categorical variables were expressed as median (interquartile spacing) and absolute values (percentages), respectively. NHANES 2001–2006 correlation coefficients of serum and dietary levels of carotenoids in adults were determined by utilizing the Spearman correlation method. The interquartile spacing of serum levels of carotenoids was determined according to the distribution of the target population. The data were log-transformed and divided into quartiles, wherein the lowest quartile was regarded as the benchmark. The inter-group differences of categorical variables, non-normally distributed continuous variables, and normally distributed continuous variables were assessed by utilizing one-way ANOVA test, Kruskal-Wallis test and χ2 test, respectively.

Two statistical models were developed in this study. Specifically, Model 1 was adjusted for gender (male or female), age (18–39, 40–59, or ≥60), and race; Model 2 was adjusted for Model 1 and education level, household income (income-to-poverty ratio ≤ 1.0, 1.1–3.0, or >3.0), smoking history, drinking history, BMI (<25.0 kg/m^2^, 25.0–29.9 kg/m^2^, or > 29.9 kg/m^2^), energy intake level (low, adequate, or high), physical activity, hypertension, diabetes, hypercholesterolemia, and supplement use (yes or no).

The correlation of the total carotenoid levels with CVD-related outcomes was assessed by using weighted quantile sum (WQS) regression. Herein, a weight in an index, which indicates the contribution to the overall protective association, was assigned to each carotenoid. In the model, 40% of the data was assigned to the training set and 60% to the validation set, meanwhile the training set was bootstrapped 1,000 times to maximize the likelihood function of the linear model. The proposed WQS regression model was exposed to adjustment for the factors mentioned above. Serum level of one single carotenoid ≥0.1 was regarded as a significant contributor. The dose–response correlation of carotenoids in serum and the prevalence of CVD was explored by using a restricted cubic spline (RCS) regression model with different percentiles (10th, 50th, and 90th). ANOVA was used to clarify the nonlinearity. R software was employed for all statistical analyses. *p* < 0.05 (two-sided) denoted statistical significance.

## 3. Results

### 3.1. Baseline features of the participants

50.77% of the 12,424 participants enrolled were male and the overall CVD-weighted prevalence was 12.9%. Table 1 shows the survey-weighted health and sociodemographic features of the respondents.

**Table 1:** Survey-weighted features of participants with available data on serum levels of all carotenoids involved in NHANES 2001–2006 (*n* = 12,424).

Characteristics	*N* (%)
Age, %	
18–39 years old	4,023 (32.38)
40–59 years old	3,972 (31.97)
≥60 years old	4,429 (35.65)
Male, %	6,308 (50.77)
Race/ethnicity, %	
Mexican American	2,510 (20.20)
Other Hispanic	431 (3.47)
Non-Hispanic White	6,534 (52.59)
Non-Hispanic Black	2,485 (20.00)
Others	464 (3.73)
Education level, %	
Below high school	3,622 (29.15)
High school	3,018 (24.29)
Above high school	5,784 (46.56)
Family income-to-poverty ratio, %	
≤1.0	2,156 (17.83)
1.1–3.0	4,935 (40.82)
>3.0	5,000 (41.35)
Smoking history, %	
Never smoker	6,238 (50.21)
Former smoker	3,322 (26.74)
Current smoker	2,864 (23.05)
Drinking history, %	
Nondrinker	2,921 (23.51)
Low-to-moderate drinker	8,460 (68.09)
Heavy drinker	1,043 (8.40)
Body mass index, kg/m^2^	
<25.0	3,839 (30.90)
25.0–29.9	4,460 (35.90)
>29.9	4,125 (33.20)
Physical activity, %	
Inactive	3,476 (27.98)
Insufficiently active	6,090 (49.02)
Active	2,858 (23)
Energy intake, kcal/day	
Low	5,240 (42.18)
Adequate	4,539 (36.53)
High	2,645 (21.29)
Hypercholesterolemia, %	4,539 (34.35)
Hypertension, %	4,141 (33.33)
Diabetes, %	1,302 (10.48)
Carotenoids supplement use, %	3,512 (28.27)
Congestive heart failure, %	428 (3.44)
Coronary heart disease, %	587 (4.72)
Angina, %	467 (3.76)
Heart attack, %	603 (4.85)
Stroke, %	475 (3.82)
Cardiovascular disease, %	1,527 (12.29)

Data are expressed in the form of *n* (percentage); sampling weights are used to calculate demographic data; n and percentages denote the sample number and the survey-weighted value, respectively.

### 3.2. Distribution and concentration of serum levels and dietary intake of carotenoids

The baseline distribution and serum levels of the carotenoids involved are summarized in Supplementary Table S1. As observed, the serum levels of β-carotene, lutein/zeaxanthin, lycopene, β-cryptoxanthin, and α-carotene were 23.56, 19.62, 15.75, 9.38, and 4.41 μg/dl, respectively. The total concentration of serum carotenoids was 72.71 μg/dl. The mean concentrations of dietary lycopene, β-carotene, lutein and zeaxanthin, α-carotene, and β-cryptoxanthin were 6,216.10, 1,931.16, 1,386.18, 372.00, and 137.90 μg/dl, respectively. The Spearman correlation coefficients of carotenoids in serum were 0.19–0.76, indicating a moderate to strong correlation. Among them, α-carotene and β-carotene showed the strongest correlation (r = 0.76; see Supplementary Figure S2).

### 3.3. Association of carotenoids in serum with prevalence of CVD

The serum levels of carotenoids involved were categorized into quartiles. Herein, the minimum quartile was regarded as the benchmark, and its associations with CVD prevalence were assessed. According to Table 2, carotenoids except for β-carotene were negatively related to CVD prevalence. Model 1 was generated after adjusting for age, sex, and race, and all carotenoids showed a negative association with the prevalence of CVD compared to the benchmark. Model 2 was adjusted for all other factors mentioned above on the basis of Model 1. In the Model 2, lutein/zeaxanthin (OR = 0.69, 95% CI: 0.54–0.86, *p* < 0.001) and lycopene (OR = 0.53, 95% CI: 0.41 ~ 0.67, *p* < 0.001) were significantly and negatively related to CVD prevalence, compared to the benchmark. However, the negative associations of β-cryptoxanthin, β-carotene, and α-carotene with CVD were attenuated in Model 2.

**Table 2:** ORs (95% CIs) of CVD prevalence among adults in NHANES 2001–2006.

	Carotenoids in serum (μg/dL)				
	Quartile 1	Quartile 2	Quartile 3	Quartile 4	*P*_trend_
α-Carotene					
Crude	1 [xx]	0.90 (0.75–1.09)	0.82 (0.67–1.00)	0.61 (0.49–0.75)	<0.001
Model 1	1 [xx]	0.72 (0.59–0.87)	0.57 (0.46–0.69)	0.40 (0.32–0.49)	<0.001
Model 2	1 [xx]	0.75 (0.60–0.93)	0.72 (0.58–0.88)	0.61 (0.47–0.79)	0.001
β-Carotene					
Crude	1 [xx]	0.96 (0.80–1.16)	1.03 (0.89–1.18)	0.94 (0.79–1.12)	0.616
Model 1	1 [xx]	0.80 (0.65–0.97)	0.65 (0.55–0.76)	0.46 (0.39–0.55)	<0.001
Model 2	1 [xx]	0.87 (0.70–1.08)	0.84 (0.70–1.02)	0.73 (0.59–0.91)	0.008
β-Cryptoxanthin					
Crude	1 [xx]	0.70 (0.60–0.80)	0.60 (0.53–0.69)	0.49 (0.40–0.61)	<0.001
Model 1	1 [xx]	0.72 (0.61–0.85)	0.59 (0.51–0.68)	0.43 (0.34–0.54)	<0.001
Model 2	1 [xx]	0.84 (0.68–1.03)	0.74 (0.62–0.88)	0.67 (0.50–0.89)	0.002
Lutein/zeaxanthin					
Crude	1 [xx]	0.79 (0.67–0.93)	0.72 (0.61–0.84)	0.76 (0.65–0.89)	<0.001
Model 1	1 [xx]	0.69 (0.56–0.85)	0.55 (0.46–0.65)	0.48 (0.39–0.58)	<0.001
Model 2	1 [xx]	0.77 (0.61–0.98)	0.66 (0.52–0.82)	0.69 (0.54–0.86)	<0.001
Lycopene					
Crude	1 [xx]	0.46 (0.38–0.56)	0.34 (0.29–0.40)	0.23 (0.18–0.29)	<0.001
Model 1	1 [xx]	0.66 (0.54–0.79)	0.57 (0.47–0.68)	0.41 (0.33–0.52)	<0.001
Model 2	1 [Reference]	0.76 (0.64–0.91)	0.68 (0.56–0.82)	0.53 (0.41–0.67)	<0.001
Total carotenoids					
Crude	1 [xx]	0.63 (0.53–0.75)	0.58 (0.48–0.69)	0.46 (0.38–0.54)	<0.001
Model 1	1 [xx]	0.65 (0.54–0.78)	0.58 (0.48–0.71)	0.36 (0.30–0.43)	<0.001
Model 2	1 [xx]	0.73 (0.61–0.87)	0.75 (0.60–0.93)	0.55 (0.45–0.68)	<0.001

Model 1 was adjusted for age (18–39, 40–59, or ≥ 60), sex (male or female), and race (Mexican American, Other Hispanic, Non-Hispanic White, Non-Hispanic Black or Other);

Model 2 was adjusted for Model 1 plus education level (below high school, high school, or above high school), family income-to-poverty ratio (≤1.0, 1.1–3.0, or > 3.0), smoking status (never smoker, former smoker, or current smoker), drinking status (nondrinker, low-to-moderate drinker, or heavy drinker), BMI (<25.0 kg/m^2^, 25.0–29.9 kg/m^2^, or >29.9 kg/m^2^), energy intake levels (low, adequate, or high), physical activity (inactive, insufficiently active, or active), hypercholesterolemia (yes or no), diabetes (yes or no), hypertension (yes or no), and supplement use (yes or no).

### 3.4. WQS regression analysis of the negative correlation of the total serum carotenoids with total and specific CVD

**Figure 1:**
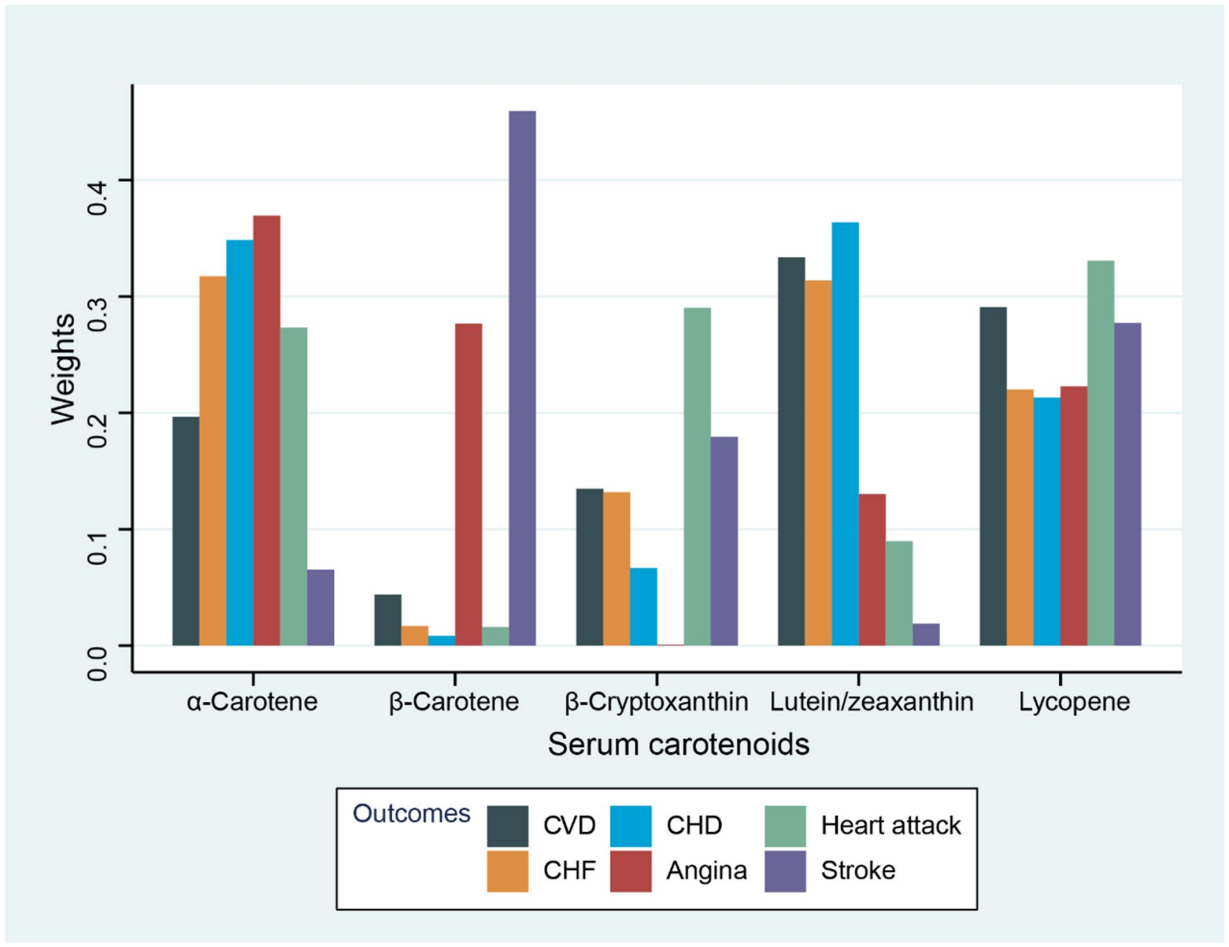
Weights from WQS regression for the serum carotenoids and CVD-related outcomes with adjustment for gender, race, age, household income, drinking history, education level, BMI, energy intake level, smoking history, physical activity, hyperlipidemia, diabetes, and hypertension.

The association of total concentrations of serum carotenoids with various CVD-related outcomes was assessed by using WQS regression (Table 3). As observed, the combination of serum carotenoids was negatively related to the prevalence of total CVD (OR = 0.88, 95% CI: 0.85–0.92, *p* < 0.001). In addition, the combination of serum carotenoids was also associated with the prevalence of specific CVD, with multivariable-adjusted ORs of 0.91 (95% CI: 0.85–0.98, *p* = 0.010) for CHF; 0.91 (95% CI, 0.86–0.96, *p* = 0.001) for CHD; 0.93 (95% CI, 0.87–0.99, *p* = 0.034) for angina; 0.88 (95% CI, 0.83–0.94, *p* < 0.001) for heart attack; and 0.89 (95% CI, 0.84–0.95, *p* < 0.001) for stroke. Figure 1 shows the weights of each serum carotenoid in the overall protective effects on different CVDs.

**Table 3:** WQS regression model used for assessment of the correlation of the serum carotenoids with total and specific CVD.

	OR	95% CI	*p* value
Total CVD	0.88	0.85–0.92	<0.001
Specific CVD			
Congestive heart failure	0.91	0.85–0.98	0.010
Coronary heart disease	0.91	0.86–0.96	0.001
Angina	0.93	0.87–0.99	0.034
Heart attack	0.88	0.83–0.94	<0.001
Stroke	0.89	0.84–0.95	<0.001

WQS, weighted quantile sum; CI, confidence interval; and OR, odds ratio. WQS regression model was exposed to adjustment for gender, race, age, household income, education level, drinking history, smoking history, BMI, physical activity, energy intake levels, hypercholesterolemia, hypertension, and diabetes.

### 3.5. RCS analysis of the correlation of CVD with serum carotenoids

**Figure 2:**
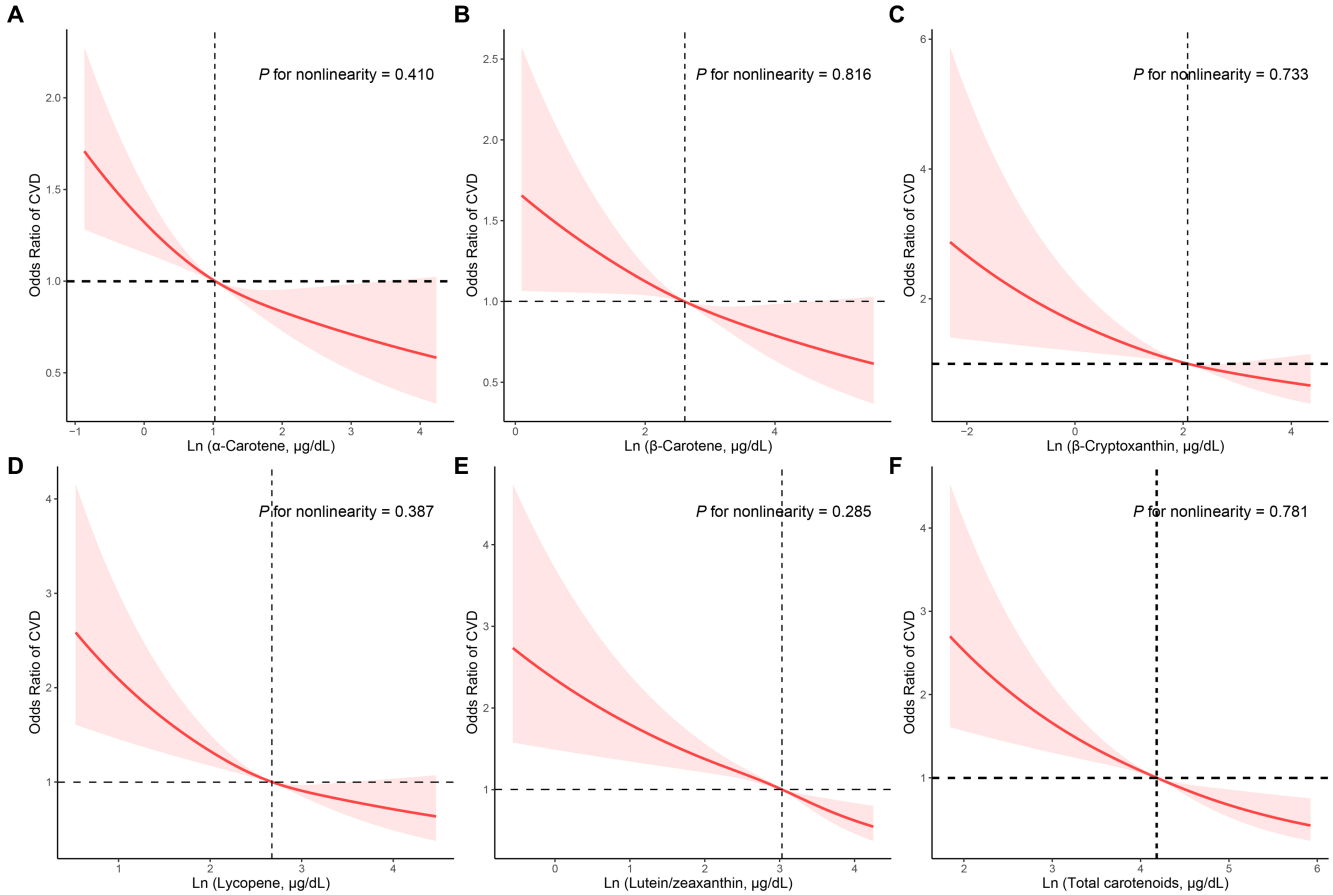
RCS analysis with multivariate adjustment of the correlation of the carotenoids and CVD prevalence. **(A)** α-Carotene, **(B)** β-Carotene, **(C)** β-Cryptoxanthin, **(D)** Lycopene, **(E)** Lutein/zeaxanthin, **(F)** Total carotenoids.

The dose–response correlation of the five carotenoids and total carotenoids with CVD prevalence was visualized by utilizing RCS regression with multivariate adjustment (Figure 2). All carotenoids and total carotenoids were negatively and linearly related to the prevalence of CVD, respectively (α-carotene: *p* was 0.410, Figure 2A; β-carotene: *p* was 0.816, Figure 2B; β-cryptoxanthin: *p* was 0.733, Figure 2C; lycopene: *p* was 0.387, Figure 2D; lutein/zeaxanthin: *p* was 0.285, Figure 2E; total carotenoids: *p* was 0.781, Figure 2F).

## 4. Discussion

The correlation of serum levels of carotenoids and CVD prevalence was explored. The results exhibited that the serum level of total carotenoids was negatively related to CVD. Among the five carotenoids, β-cryptoxanthin and lycopene were negatively related to the prevalence of CVD. In addition, WQS analysis showed that serum levels of total carotenoids were related to myocardial infarction and stroke in CVD. Additionally, RCS analysis showed a linear negative correlation of serum carotenoids with CVD.

Higher dietary intake or blood carotenoid concentrations were associated with a reduced risk of cardiovascular disease (18). Previous studies have shown that the cardiovascular risk can be reduced by increasing the intake of β-carotene or serum/plasma levels of β-carotene (19). A population-based study involving middle-aged male participants shows that the risk of CHF is negatively correlated with the serum level of β-carotene (20). For α-carotene, a high serum level of α-carotene can relieve CVD (21). A stratified analysis revealed that an elevated serum level of α-carotene can trigger beneficial changes in the variability of heart rate of adults (22). As an antioxidant (23), β-Cryptoxanthin may have anti-cancer effects (24) and reduce the risk of osteoporosis (25). However, few studies discussing the correlation of β-Cryptoxanthin with CVD have been reported. A cross-sectional analysis from Mikkabi concluded that the serum level of b-cryptoxanthin is inversely proportional to the risk of atherosclerosis (26).

However, the effect of carotenoid supplements on cardiovascular disease has shown different results. A recent meta-analysis suggests that the incidence of major CVD is not related to β-carotene supplementation (27). Meanwhile, the incidence of coronary atherosclerotic heart disease has no significant association with β-carotene (12). For lycopene, most of the research is concentrated on lycopene supplements. It has been demonstrated that lycopene supplementation can deliver significant reductions in LDL cholesterol (28). In a human dietary intervention study, reductions in lipid and LDL oxidation were observed after 1 week of lycopene supplementation (10, 29). Additionally, lycopene improves the function of high-density lipoprotein (HDL) (30, 31). Lycopene in serum has beneficial effects on CHD, which is consistent with the present study, demonstrating that lycopene also exhibited beneficial effects on CHF, angina, heart attack, and stroke (32).

The present study also clarifies the role of β-Cryptoxanthin in CVD. In addition to CHD, β-Cryptoxanthin has a great effect on heart attack. However, the protection of β-Cryptoxanthin on CVD was not enhanced in Model 2 adjusted for household income, education level, and smoking history. Meanwhile, lutein/zeaxanthin may have beneficial effects on CVD by alleviating chronic inflammation in CVD patients (33). Indeed, the risks of coronary heart disease and stroke are inversely proportional to the intake or concentration of lutein/zeaxanthin (34) as lutein/zeaxanthin can improve vascular tone and endothelial function (35). Additionally, in a cohort study based on older adults, serum lutein/zeaxanthin levels were significantly associated with telomere length, which is associated with increasing age and age-related diseases such as stroke, diabetes, cardiovascular disease and cancer (36). In the present study, however, stroke had no significant correlation with lutein/zeaxanthin.

Oxidative stress (OS) plays a key role in CVD. It is also related to various abnormalities, including systemic inflammation, immune cell activation, sympathetic nervous system excitation, renal dysfunction, cardiovascular remodeling, vascular dysfunction, and endothelial damage, by triggering redox signaling of reactive oxygen species (ROS) (37, 38). As an antioxidant, carotenoids can inhibit peroxidation, eliminate free radicals, scavenge lipid peroxyl radicals, and reduce ROS-induced damage (39). Additionally, high serum levels of carotenoids are related to low thickness of carotid intima-media, which has preventive implications for CHD (40).

Despite that the protective effect of carotenoids against CVD has been demonstrated, the specific contribution of each carotenoid to this effect remains unclear. This study revealed that serum carotenoids protect human body from CVD, with the protective effects of lutein and lycopene being well established. However, the protective effects of β-cryptoxanthin, β-carotene and α-carotene on CVD were negligible after model adjustment. Indeed, the intakes of beta-carotene and lycopene are negatively related to death, stroke, and coronary heart disease, while total carotenoid, lycopene, β-cryptoxanthin, β-carotene, and α-carotene are negatively related to all-cause mortality from CVD (18). This study indicated that the five carotenoids and total serum carotenoids have a negative linear correlation with the prevalence of CVD. WQS regression analysis indicated negative correlations of total serum carotenoids with specific CVDs, especially stroke and heart attack. However, the findings remain to be confirmed by future studies.

This study presents several advantages. First, it serves as a preliminary assessment of the overall protective effect of different carotenoids against CVDs. The protective effects of carotene, lycopene, and lutein on CVD were confirmed, while the protective effects of β-carotene, α-carotene and β- cryptoxanthin were negligible after as adjustment for education level and poverty. Second, this study was based on a large yet representative sample and the results were statistically significant. Third, WQS regression was employed to preserve statistical effects and avoid unstable regression coefficients. In addition, this approach reveals correlations between exposures and exposure-outcomes.

Nevertheless, several limitations are also noted. Due to the observational study design, no causal relationships could be determined. In other words, the causal relationship between serum levels of carotenoids and CVD prevalence has not been fully understood. Additionally, other dietary or environmental factors (e.g., artificial sweeteners, vitamin D, dietary fiber, and heavy metals) may still have influences on the conclusions even after model adjustment as carotenoids are mainly obtained from fruits and vegetables (41–44). The limitations of the WQS regression analysis may also affect the conclusions as the all-positive and all-negative dependent variables have the same effects on exposure.

## 5. Conclusion

With adjustment for age, race, gender, poverty, education level, smoking history, alcohol history, BMI, energy intake level, physical activity, hyperlipidemia, diabetes, and hypertension supplement, serum levels of carotenoids (total, lycopene, α-carotene, lutein/zeaxanthin, β-carotene, and β-cryptoxanthin) were negatively associated with the prevalence of CVD. However, the effect of β-carotene on CVD remains unclear. Additionally, the five serum carotenoids had inverse linear correlations with CVD prevalence. There was a strong negative correlation between serum concentrations of lycopene and the prevalence of CVDs (e.g., CHF, CHD, angina, heart attack, and stroke). Nevertheless, future studies clarifying the complex interactions between different carotenoids in serum, as well as their effects on CVDs, are of great significance.

## Data availability statement

The original contributions presented in the study are included in the article/Supplementary material, further inquiries can be directed to the corresponding author.

## Ethics statement

Ethical review and approval was not required for the study on human participants in accordance with the local legislation and institutional requirements. Written informed consent from the patients/participants or patients/participants' legal guardian/next of kin was not required to participate in this study in accordance with the national legislation and the institutional requirements.

## Author contributions

MW designed the present study and performed the data analysis. RT and DD contributed equally to the writing of this article. RZ and YQ critically revised and edited the manuscript for important intellectual content. All authors contributed to the article and approved the submitted version.

## Funding

This work was supported by the Young Talent Development plan of Changzhou Health Commission (CZQM2020034 and CZQM2020004), Young talents Science and technology project of Changzhou Health Commission (QN201913), and Social Development Projects of Changzhou Science and Technology Bureau (CE20205039).

## Conflict of interest

The authors declare that the research was conducted in the absence of any commercial or financial relationships that could be construed as a potential conflict of interest.

## Publisher’s note

All claims expressed in this article are solely those of the authors and do not necessarily represent those of their affiliated organizations, or those of the publisher, the editors and the reviewers. Any product that may be evaluated in this article, or claim that may be made by its manufacturer, is not guaranteed or endorsed by the publisher.
