# Supplementary material for: The protective effect of serum carotenoids on cardiovascular disease: a cross-sectional study from the general US adult population
Source: Front Nutr. 2023 Jul 12;10:1154239. doi: 10.3389/fnut.2023.1154239 (PMC10368866; doi:10.3389/fnut.2023.1154239)
Supplement: Supplementary file 1 [file Data_Sheet_1.docx]

**Online Supplementary Material**

**Associations of Serum Carotenoids with Cardiovascular Disease for the General Adult Population** Authors：Min Wang, Renzhe Tang, Rui Zhou, Yongxiang Qian, Bin Wang, Dongmei Di

**Figure S1.** Flowchart of the study participants.

**Table S1.** Distributions and concentrations of serum carotenoids and dietary carotenoid intake levels among adults in NHANES 2001–2006.

**Figure S2.** Pairwise Spearman correlation coefficients among serum and dietary carotenoids among adults in NHANES 2001–2006.

**Table S2.** ORs (95% CIs) of the prevalence of specific cardiovascular disease (CVD) according to quartiles of serum carotenoids levels among adults in NHANES 2001–2006.

**Table S3.** ORs (95% CIs) of the prevalence of cardiovascular disease (CVD) according to quintiles of serum carotenoids levels among adults in NHANES 2001–2006.

**Table S4.** ORs (95% CIs) of the prevalence of cardiovascular disease (CVD) according to quartiles of serum carotenoids levels with further adjustment of respective dietary carotenoid intakes among adults in NHANES 2001–2006.

**Table S5.** ORs (95% CIs) of the prevalence of cardiovascular disease (CVD) according to quintiles of dietary carotenoid intake levels among adults in NHANES 2001–2006.

**
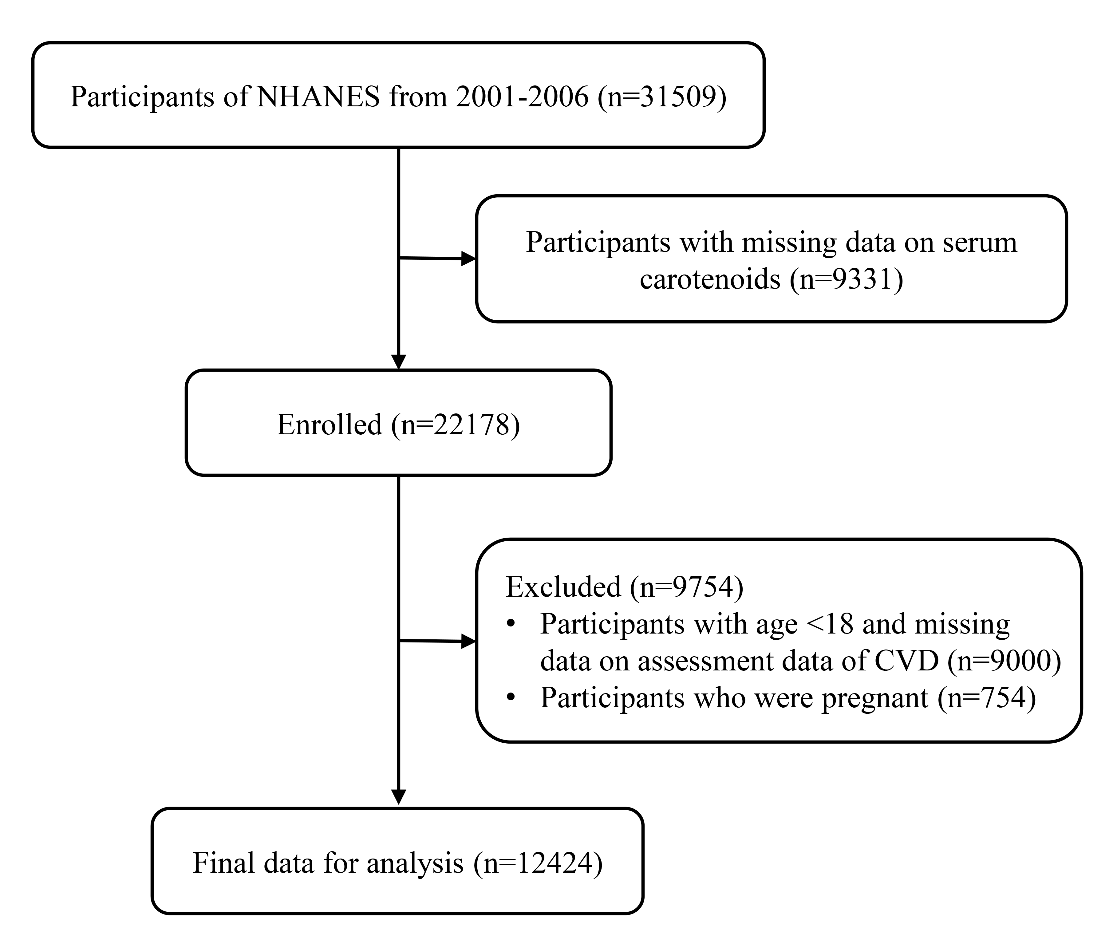
**

**Figure S1.** Flowchart of the study participants

**Table S1.** Distributions and concentrations of serum carotenoids and dietary carotenoid intake levels among adults in NHANES 2001–2006.

|  | Mean | | GM | 5^th^ | | 25^th^ | | 50^th^ | 75^th^ | | 95^th^ |
| --- | --- | --- | --- | --- | --- | --- | --- | --- | --- | --- | --- |
| ***Serum carotenoids*** | |  | | |  | |  | | |  | |
| α-Carotene, μg/dL | 4.41 | | 2.78 | 0.50 | | 1.46 | | 2.70 | 5.11 | | 13.40 |
| β-Carotene, μg/dL | 19.62 | | 13.58 | 3.79 | | 7.69 | | 12.89 | 23.30 | | 56.40 |
| β-Cryptoxanthin, μg/dL | 9.38 | | 7.31 | 2.40 | | 4.67 | | 7.27 | 11.50 | | 23.40 |
| Lycopene, μg/dL | 15.75 | | 13.78 | 5.94 | | 9.90 | | 13.90 | 19.49 | | 31.70 |
| Lutein/zeaxanthin, μg/dL | 23.56 | | 20.54 | 7.20 | | 15.40 | | 22.40 | 30.34 | | 43.80 |
| Total carotenoids, ug/dL | 72.71 | | 64.18 | 28.39 | | 47.60 | | 64.40 | 88.00 | | 143.29 |
| ***Dietary carotenoids*** | |  | | |  | |  | | |  | |
| α-Carotene, μg/day | 372.00 | | 45.25 | 0.00 | | 12.00 | | 45.00 | 204.00 | | 1978.00 |
| β-Carotene, μg/day | 1931.16 | | 721.73 | 63.00 | | 292.00 | | 717.00 | 2050.00 | | 7560.00 |
| β-Cryptoxanthin, μg/day | 137.90 | | 26.89 | 0.00 | | 6.00 | | 26.00 | 159.00 | | 620.00 |
| Lycopene, μg/day | 6216.10 | | 367.02 | 0.00 | | 1.00 | | 1798.00 | 7411.00 | | 27180.00 |
| Lutein/zeaxanthin, μg/day | 1386.18 | | 636.89 | 75.00 | | 317.00 | | 676.00 | 1368.00 | | 4630.00 |

GM, geometric mean; 5^th^, 5th percentile; 25^th^, 25th percentile; 50^th^, 50th percentile; 75^th^, 75th percentile; 95^th^, 95th percentile.

**
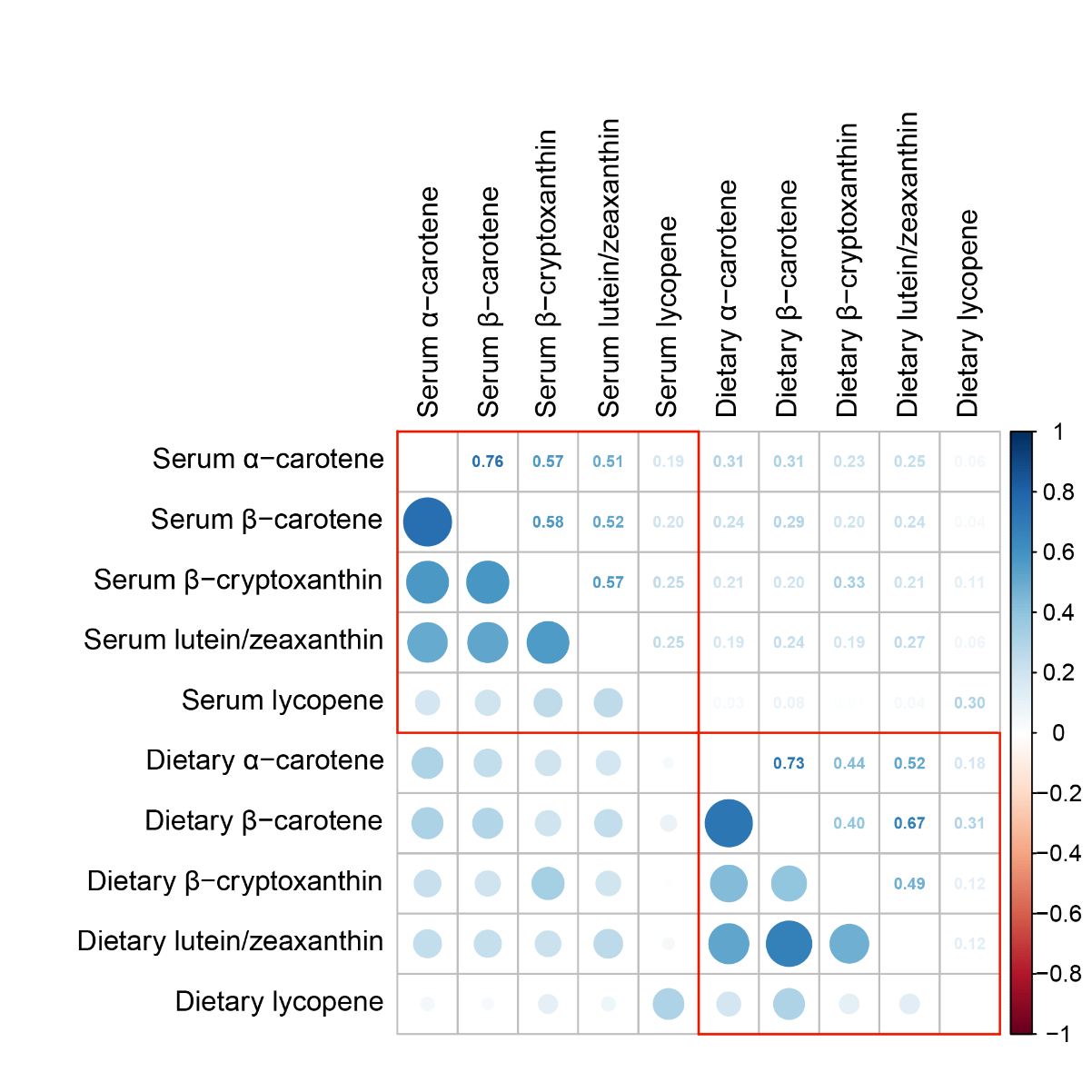
Figure S2.** Pairwise Spearman correlation coefficients among serum and dietary carotenoids among adults in NHANES 2001–2006.

**Table S2.** ORs (95% CIs) of the prevalence of specific cardiovascular disease (CVD) according to quartiles of serum carotenoids levels among adults in NHANES 2001–2006.

|  | Serum carotenoids, μg/dL | | | |  |
| --- | --- | --- | --- | --- | --- |
|  | Quartile 1 | Quartile 2 | Quartile 3 | Quartile 4 | *P* _trend_ |
| ***Congestive heart failure*** | |  |  |  |  |
| α-Carotene | 1 [Reference] | 0.52 (0.37-0.74) | 0.52 (0.33-0.81) | 0.58 (0.39-0.84) | 0.012 |
| β-Carotene | 1 [Reference] | 0.67 (0.46-0.98) | 0.64 (0.41-0.99) | 0.81 (0.52-1.26) | 0.293 |
| β-Cryptoxanthin | 1 [Reference] | 0.95 (0.69-1.30) | 0.69 (0.44-1.09) | 0.67 (0.42-1.07) | 0.040 |
| Lutein/zeaxanthin | 1 [Reference] | 0.81 (0.58-1.12) | 0.47 (0.34-0.67) | 0.54 (0.36-0.81) | <0.001 |
| Lycopene | 1 [Reference] | 0.78 (0.542-1.13) | 0.52 (0.38-0.71) | 0.47 (0.29-0.74) | <0.001 |
| Total carotenoids | 1 [Reference] | 0.53 (0.36-0.77) | 0.51 (0.32-0.82) | 0.60 (0.41-0.87) | 0.005 |
| ***Coronary heart disease*** | |  |  |  |  |
| α-Carotene | 1 [Reference] | 0.88 (0.63-1.22) | 0.89 (0.68-1.16) | 0.60 (0.43-0.84) | 0.008 |
| β-Carotene | 1 [Reference] | 1.17 (0.85-1.61) | 0.94 (0.64-1.38) | 0.80 (0.57-1.12) | 0.101 |
| β-Cryptoxanthin | 1 [Reference] | 0.82 (0.59-1.13) | 0.73 (0.56-0.94) | 0.63 (0.45-0.90) | 0.004 |
| Lutein/zeaxanthin | 1 [Reference] | 0.69 (0.51-0.93) | 0.67 (0.47-0.96) | 0.64 (0.43-0.94) | 0.021 |
| Lycopene | 1 [Reference] | 0.94 (0.69-1.29) | 0.81 (0.61-1.09) | 0.61 (0.43-0.88) | 0.004 |
| Total carotenoids | 1 [Reference] | 0.83 (0.64-1.07) | 0.77 (0.51-1.15) | 0.62 (0.47-0.82) | 0.004 |
| ***Angina*** |  |  |  |  |  |
| α-Carotene | 1 [Reference] | 0.83 (0.58-1.19) | 0.88 (0.59-1.31) | 0.72 (0.47-1.10) | 0.166 |
| β-Carotene | 1 [Reference] | 1.04 (0.71-1.50) | 0.88 (0.59-1.29) | 0.74 (0.49-1.13) | 0.099 |
| β-Cryptoxanthin | 1 [Reference] | 1.16 (0.74-1.80) | 1.04 (0.68-1.57) | 0.97 (0.61-1.54) | 0.887 |
| Lutein/zeaxanthin | 1 [Reference] | 0.68 (0.48-0.97) | 0.67 (0.43-1.06) | 0.59 (0.39-0.88) | 0.017 |
| Lycopene | 1 [Reference] | 0.80 (0.60-1.06) | 0.90 (0.60-1.34) | 0.75 (0.51-1.11) | 0.215 |
| Total carotenoids | 1 [Reference] | 0.68 (0.44-1.03) | 0.99 (0.66-1.50) | 0.62 (0.42-0.92) | 0.118 |
| ***Heart attack*** |  |  |  |  |  |
| α-Carotene | 1 [Reference] | 0.75 (0.54-1.04) | 0.79 (0.60-1.04) | 0.62 (0.43-0.90) | 0.017 |
| β-Carotene | 1 [Reference] | 0.78 (0.55-1.09) | 0.72 (0.53-0.98) | 0.58 (0.40-0.85) | 0.007 |
| β-Cryptoxanthin | 1 [Reference] | 0.92 (0.68-1.25) | 0.70 (0.54-0.90) | 0.73 (0.51-1.06) | 0.016 |
| Lutein/zeaxanthin | 1 [Reference] | 0.66 (0.47-0.93) | 0.72 (0.49-1.04) | 0.69 (0.49-0.98) | 0.070 |
| Lycopene | 1 [Reference] | 0.77 (0.60-0.99) | 0.69 (0.50-0.95) | 0.51 (0.33-0.79) | 0.004 |
| Total carotenoids | 1 [Reference] | 0.73 (0.55-0.96) | 0.67 (0.47-0.97) | 0.55 (0.41-0.73) | <0.001 |
| ***Stroke*** |  |  |  |  |  |
| α-Carotene | 1 [Reference] | 0.56 (0.42-0.73) | 0.61 (0.40-0.91) | 0.59 (0.37-0.92) | 0.032 |
| β-Carotene | 1 [Reference] | 0.64 (0.45-0.90) | 0.65 (0.44-0.95) | 0.66 (0.45-0.97) | 0.048 |
| β-Cryptoxanthin | 1 [Reference] | 0.86 (0.64-1.14) | 0.78 (0.55-1.10) | 0.76 (0.50-1.18) | 0.137 |
| Lutein/zeaxanthin | 1 [Reference] | 0.77 (0.50-1.19) | 0.74 (0.51-1.07) | 0.69 (0.49-0.98) | 0.041 |
| Lycopene | 1 [Reference] | 0.64 (0.45-0.92) | 0.69 (0.52-0.92) | 0.45 (0.28-0.73) | <0.001 |
| Total carotenoids | 1 [Reference] | 0.69 (0.52-0.91) | 0.59 (0.44-0.80) | 0.55 (0.37-0.82) | <0.001 |

Model 1 was adjusted for age (18-39, 40-59, or ≥60), sex (male or female), and race (Mexican American, Other Hispanic, Non-Hispanic White, Non-Hispanic Black or Other);

Model 2 was adjusted for Model 1 plus education level (below high school, high school, or above high school), family income-to-poverty ratio (≤1.0, 1.1–3.0, or >3.0), smoking status (never smoker, former smoker, or current smoker), drinking status (nondrinker, low-to-moderate drinker, or heavy drinker), BMI (<25.0, 25.0-29.9, or >29.9), energy intake levels (low, adequate, or high), physical activity (inactive, insufficiently active, or active), hypercholesterolemia (yes or no), diabetes (yes or no), hypertension (yes or no), and supplement use (yes or no).

**Table S3.** ORs (95% CIs) of the prevalence of cardiovascular disease (CVD) according to quintiles of serum carotenoids levels among adults in NHANES 2001–2006.

|  | Serum carotenoids, μg/dL | | | | |  |
| --- | --- | --- | --- | --- | --- | --- |
|  | Quartile 1 | Quartile 2 | Quartile 3 | Quartile 4 | Quartile 5 | *P* _trend_ |
| **α-Carotene** |  |  |  |  |  |  |
| Crude | 1 [Reference] | 0.91 (0.74-1.12) | 0.82 (0.63-1.07) | 0.76 (0.63-0.91) | 0.55 (0.43-0.70) | <0.001 |
| Model 1 | 1 [Reference] | 0.76 (0.60-0.95) | 0.59 (0.45-0.76) | 0.49 (0.41-0.59) | 0.35 (0.27-0.46) | <0.001 |
| Model 2 | 1 [Reference] | 0.79 (0.62-1.02) | 0.68 (0.52-0.88) | 0.63 (0.51-0.78) | 0.56 (0.42-0.75) | <0.001 |
| **β-Carotene** |  |  |  |  |  |  |
| Crude | 1 [Reference] | 0.86 (0.71-1.05) | 1.04 (0.81-1.33) | 0.98 (0.83-1.16) | 0.89 (0.72-1.09) | 0.593 |
| Model 1 | 1 [Reference] | 0.78 (0.62-0.97) | 0.74 (0.58-0.96) | 0.583 (0.49-0.69) | 0.43 (0.35-0.52) | <0.001 |
| Model 2 | 1 [Reference] | 0.85 (0.64-1.12) | 0.89 (0.68-1.16) | 0.80 (0.66-0.97) | 0.69 (0.55-0.87) | 0.002 |
| **β-Cryptoxanthin** | |  |  |  |  |  |
| Crude | 1 [Reference] | 0.68 (0.57-0.81) | 0.65 (0.55-0.77) | 0.62 (0.52-0.72) | 0.46 (0.36-0.59) | <0.001 |
| Model 1 | 1 [Reference] | 0.72 (0.60-0.87) | 0.67 (0.55-0.82) | 0.58 (0.49-0.69) | 0.40 (0.30-0.52) | <0.001 |
| Model 2 | 1 [Reference] | 0.81 (0.65-0.99) | 0.81 (0.65-1.00) | 0.79 (0.64-0.98) | 0.63 (0.45-0.87) | 0.007 |
| **Lutein/zeaxanthin** | |  |  |  |  |  |
| Crude | 1 [Reference] | 0.87 (0.74-1.03) | 0.72 (0.60-0.87) | 0.73 (0.60-0.89) | 0.73 (0.61-0.87) | <0.001 |
| Model 1 | 1 [Reference] | 0.80 (0.66-0.98) | 0.60 (0.49-0.72) | 0.54 (0.44-0.66) | 0.45 (0.37-0.55) | <0.001 |
| Model 2 | 1 [Reference] | 0.85 (0.68-1.08) | 0.70 (0.55-0.89) | 0.70 (0.55-0.88) | 0.67 (0.53-0.85) | 0.001 |
| **Lycopene** |  |  |  |  |  |  |
| Crude | 1 [Reference] | 0.49 (0.39-0.61) | 0.32 (0.27-0.39) | 0.29 (0.24-0.36) | 0.22 (0.17-0.27) | <0.001 |
| Model 1 | 1 [Reference] | 0.66 (0.52-0.84) | 0.52 (0.42-0.63) | 0.52 (0.42-0.64) | 0.41 (0.32-0.51) | <0.001 |
| Model 2 | 1 [Reference] | 0.76 (0.59-0.98) | 0.60 (0.49-0.75) | 0.64 (0.50-0.81) | 0.53 (0.42-0.66) | <0.001 |
| **Total carotenoids** | |  |  |  |  |  |
| Crude | 1 [Reference] | 0.57 (0.47-0.69) | 0.55 (0.45-0.67) | 0.48 (0.40-0.58) | 0.38 (0.31-0.46) | <0.001 |
| Model 1 | 1 [Reference] | 0.60 (0.49-0.73) | 0.59 (0.48-0.73) | 0.47 (0.39-0.56) | 0.29 (0.23-0.37) | <0.001 |
| Model 2 | 1 [Reference] | 0.63 (0.49-0.81) | 0.72 (0.57-0.90) | 0.62 (0.49-0.77) | 0.45 (0.36-0.58) | <0.001 |

Model 1 was adjusted for age (18-39, 40-59, or ≥60), sex (male or female), and race (Mexican American, Other Hispanic, Non-Hispanic White, Non-Hispanic Black or Other);

Model 2 was adjusted for Model 1 plus education level (below high school, high school, or above high school), family income-to-poverty ratio (≤1.0, 1.1–3.0, or >3.0), smoking status (never smoker, former smoker, or current smoker), drinking status (nondrinker, low-to-moderate drinker, or heavy drinker), BMI (<25.0, 25.0-29.9, or >29.9), energy intake levels (low, adequate, or high), physical activity (inactive, insufficiently active, or active), hypercholesterolemia (yes or no), diabetes (yes or no), hypertension (yes or no), and supplement use (yes or no).

**Table S4.** ORs (95% CIs) of the prevalence of cardiovascular disease (CVD) according to quartiles of serum carotenoids levels with further adjustment of respective dietary carotenoid intakes among adults in NHANES 2001–2006.

|  | Serum carotenoids, μg/dL | | | |  |
| --- | --- | --- | --- | --- | --- |
|  | Quartile 1 | Quartile 2 | Quartile 3 | Quartile 4 | *P* _trend_ |
| **α-Carotene** | 1 [Reference] | 0.75 (0.60-0.93) | 0.72 (0.58-0.88) | 0.61 (0.47-0.79) | 0.001 |
| **β-Carotene** | 1 [Reference] | 0.87 (0.70-1.08) | 0.84 (0.70-1.02) | 0.73 (0.59-0.91) | 0.008 |
| **β-Cryptoxanthin** | 1 [Reference] | 0.84 (0.68-1.03) | 0.74 (0.62-0.88) | 0.67 (0.50-0.89) | 0.002 |
| **Lutein/zeaxanthin** | 1 [Reference] | 0.77 (0.61-0.98) | 0.66 (0.52-0.82) | 0.69 (0.54-0.86) | <0.001 |
| **Lycopene** | 1 [Reference] | 0.76 (0.64-0.91) | 0.68 (0.56-0.82) | 0.53 (0.41-0.67) | <0.001 |

Model was adjusted for age (18-39, 40-59, or ≥60), sex (male or female), race (Mexican American, Other Hispanic, Non-Hispanic White, Non-Hispanic Black or Other), education level (below high school, high school, or above high school), family income-to-poverty ratio (≤1.0, 1.1–3.0, or >3.0), smoking status (never smoker, former smoker, or current smoker), drinking status (nondrinker, low-to-moderate drinker, or heavy drinker), BMI (<25.0, 25.0-29.9, or >29.9), energy intake levels (low, adequate, or high), physical activity (inactive, insufficiently active, or active), hypercholesterolemia (yes or no), diabetes (yes or no), hypertension (yes or no), supplement use (yes or no), and respective dietary carotenoids (in quintiles).

**Table S5.** ORs (95% CIs) of the prevalence of cardiovascular disease (CVD) according to quintiles of dietary carotenoid intake levels among adults in NHANES 2001–2006.

|  | Dietary carotenoids (mg/day) | | | |  |
| --- | --- | --- | --- | --- | --- |
|  | Quartile 1 | Quartile 2 | Quartile 3 | Quartile 4 | *P* _trend_ |
| **α-Carotene** |  |  |  |  |  |
| Crude | 1 [Reference] | 0.96 (0.76-1.21) | 0.94 (0.75-1.16) | 0.91 (0.75-1.12) | 0.353 |
| Model 1 | 1 [Reference] | 0.89 (0.69-1.16) | 0.77 (0.61-0.98) | 0.67 (0.53-0.84) | <0.001 |
| Model 2 | 1 [Reference] | 0.99 (0.75-1.32) | 0.93 (0.71-1.22) | 0.80 (0.63-1.02) | 0.057 |
| **β-Carotene** |  |  |  |  |  |
| Crude | 1 [Reference] | 0.76 (0.62-0.92) | 0.83 (0.71-0.99) | 0.82 (0.68-0.98) | 0.096 |
| Model 1 | 1 [Reference] | 0.72 (0.57-0.91) | 0.70 (0.58-0.83) | 0.60 (0.48-0.74) | <0.001 |
| Model 2 | 1 [Reference] | 0.79 (0.62-1.01) | 0.81 (0.66-0.99) | 0.75 (0.59-0.96) | 0.031 |
| **β-Cryptoxanthin** | |  |  |  |  |
| Crude | 1 [Reference] | 1.07 (0.87-1.32) | 1.03 (0.86-1.24) | 1.16 (0.94-1.43) | 0.201 |
| Model 1 | 1 [Reference] | 0.93 (0.73-1.20) | 0.81 (0.66-0.99) | 0.77 (0.62-0.97) | 0.010 |
| Model 2 | 1 [Reference] | 0.97 (0.74-1.28) | 0.85 (0.66-1.09) | 0.96 (0.74-1.25) | 0.515 |
| **Lutein/zeaxanthin** | |  |  |  |  |
| Crude | 1 [Reference] | 0.89 (0.72-1.09) | 0.92 (0.77-1.10) | 0.84 (0.73-0.97) | 0.039 |
| Model 1 | 1 [Reference] | 0.73 (0.60-0.88) | 0.71 (0.58-0.86) | 0.62 (0.54-0.72) | <0.001 |
| Model 2 | 1 [Reference] | 0.78 (0.62-0.99) | 0.82 (0.65-1.04) | 0.83 (0.700-0.99) | 0.090 |
| **Lycopene** |  |  |  |  |  |
| Crude | 1 [Reference] | 0.79 (0.65-0.96) | 0.62 (0.51-0.76) | 0.58 (0.49-0.70) | <0.0001 |
| Model 1 | 1 [Reference] | 0.84 (0.68-1.02) | 0.77 (0.62-0.95) | 0.78 (0.63-0.96) | 0.012 |
| Model 2 | 1 [Reference] | 0.93 (0.74-1.17) | 0.93 (0.73-1.18) | 0.93 (0.73-1.18) | 0.496 |

Model 1 was adjusted for age (18-39, 40-59, or ≥60), sex (male or female), and race (Mexican American, Other Hispanic, Non-Hispanic White, Non-Hispanic Black or Other);

Model 2 was adjusted for Model 1 plus education level (below high school, high school, or above high school), family income-to-poverty ratio (≤1.0, 1.1–3.0, or >3.0), smoking status (never smoker, former smoker, or current smoker), drinking status (nondrinker, low-to-moderate drinker, or heavy drinker), BMI (<25.0, 25.0-29.9, or >29.9), energy intake levels (low, adequate, or high), physical activity (inactive, insufficiently active, or active), hypercholesterolemia (yes or no), diabetes (yes or no), hypertension (yes or no), and supplement use (yes or no).
